# The Development of Audio‐Tactile Spatial Integration: Unraveling Vision's Contribution

**DOI:** 10.1111/desc.70094

**Published:** 2025-11-14

**Authors:** Alessia Tonelli, Irene Senna, Maria Bianca Amadeo, Walter Setti, Nicola Domenici, Sabrina Signorini, Elena Cocchi, Giuseppina Giammari, Sandra Strazzer, Francesca Tinelli, Paola Camicione, Massimiliano Serafino, Monica Gori

**Affiliations:** ^1^ Unit for Visually Impaired People (U‐VIP) Istituto Italiano di Tecnologia Genoa Italy; ^2^ School of Psychology The University of Sydney Sydney Australia; ^3^ Department of Psychology Liverpool Hope University Liverpool UK; ^4^ Developmental Neurophthalmology Unit IRCCS Mondino Foundation Pavia Italy; ^5^ Fondazione David Chiossone Genova Italy; ^6^ Scientific Institute, IRCCS E. Medea Bosisio Parini Lecco Italy; ^7^ Department of Developmental Neuroscience IRCCS Fondazione Stella Maris Pisa Italy; ^8^ Istituto Gianna Gaslini Genova Italy

**Keywords:** audio localization, blindness, development, multisensory integration, tactile localization

## Abstract

Vision is considered the dominant sense for spatial perception. Yet, how vision contributes to its refinement in other modalities remains unclear. Consequently, we investigated the development of audio‐tactile spatial integration using a localization task in which participants had to determine the position of auditory, tactile, and audio‐tactile stimuli and the influence of visual experience in this process. We tested sighted and blind children at different ages. We found that in sighted children, tactile spatial perception stabilizes earlier than the auditory one, and optimal audio‐tactile integration is achieved only after 12 years‐of‐age. Conversely, blind children showed higher uni‐sensory precisions from a younger age, although multisensory performance exhibited minimal improvement through age. Overall, our findings suggest that optimal audio‐tactile spatial integration develops late during childhood and that vision might play a pivotal role in this process, that is, the absence of vision prompts earlier development of other sensory modalities when processing bodily stimuli.

**Summary:**

- Sighted children achieve optimal audio‐tactile spatial integration only after 12 years, aligning bimodal precision with MLE predictions in adolescence.
- Blind children show superior early uni‐modal sensory localization precision compared to sighted peers.
- Tactile precision stabilizes earlier than auditory in sighted children, whereas blind children show the opposite developmental trajectory for localization.

## 1 Introduction

To understand and navigate the world around us, it is essential to have a coherent representation of space, and vision is considered the pivotal sensory modality for its development. Multiple studies on multisensory perception have demonstrated that in spatial tasks—particularly those requiring explicit judgments about object localization—if vision conflicts with another modality (regardless of whether it is combined with touch (Badde et al. 2020) or with audio (Alais and Burr 2004)) or if visual stimuli unrelated to the task are presented (Tonelli et al. 2015), vision has a significant influence on the final percept. This evidence highlights the importance of visual input in shaping our spatial processing. However, spatial perception implies a complex interplay of sensory information that does not exclusively rely on vision but involves other senses, such as touch and audition, and their interactions.

While a substantial body of literature exists on the interaction between audition and vision (Burr and Alais 2006; Gori et al. 2012; McGovern et al. 2016) and between touch and vision (Spence et al. 2004; Gepshtein et al. 2005), little is known about the development of audio‐tactile multisensory processing in spatial representation and navigation. Some studies have shown that touch is dominant over audition in spatial tasks, producing a sort of ventriloquist effect. For instance, when audio and touch are presented with spatial displacement, and participants are asked to judge the location of the sound source, the apparent location of the sound is biased toward the tactile stimulation (Caclin et al. 2002; Bruns and Röder 2010). Also, in the spatial bisection task, it has been seen that touch can be used to recalibrate auditory representation by increasing its precision (Gori et al. 2014). However, these results can be modified by contextual factors. For example, Vercillo and Gori (2015) showed that, in an audio‐tactile bisection task, touch dominated multisensory integration due to its higher spatial precision compared to audition, but this tactile dominance was reduced when participants were required to direct their attention to the auditory stimuli.

The aforementioned studies provide a snapshot of audio‐tactile integration in adulthood, but they tell us little about how this process develops. One of the main goals of the present study is to investigate the development of this process during childhood. Specifically, we are interested in investigating whether a sensory advantage can be gained from the simultaneous presentation of audio‐tactile stimuli compared to auditory and tactile stimuli alone in a spatial task, similar to the benefits observed when vision is combined with either touch or audition. In our study, at odds with most studies in multisensory space perception, we will not directly test vision, which is the primary sense in spatial processes.

The interest in a sensory benefit followed by the presentation of multisensory stimuli stems from the so‐called “optimal cue integration theory,” according to Bayesian rules (Ernst and Bülthoff 2004). This theory posits that the brain optimally combines information from sensory cues, enhancing perceptual precision. By combining sensory cues optimally according to the maximum‐likelihood estimation model (MLE), the brain minimizes uncertainty and improves overall perceptual performance, leading to a more precise and reliable representation of the external world (Ernst and Bülthoff 2004; Ernst and Banks 2002). Nevertheless, it has been demonstrated that multisensory optimal integration is achieved relatively late during development, and before that period, there is a straightforward sensory dominance based on the sensory feature of interest (Gori et al. 2008). For example, in visual‐tactile size and orientation discrimination tasks, children start to integrate sensory information after 8 years of age. Before then, tactile dominance is observed in size perception, while visual dominance is seen for orientation, discrimination (Gori et al. 2008). When other sensory modalities are involved, such as audio and touch, integration appears to occur later in development; in fact, children before age 12 do not integrate non‐visual cues in size perception tasks (Scheller et al. 2021; Petrini et al. 2014) or in a simultaneity task (Stanley et al. 2023). In contrast, for spatial tasks, children optimally integrate audio‐visual information later at 12 years of age (Gori et al. 2012). However, this happens with visual stimuli, which are pivotal in spatial perception, and it is still unclear whether similar dynamics characterize the integration of acoustic and tactile stimuli during development. Filling this gap is the first aim of this study. Based on previous studies on audio‐tactile integration, as well as the sole study on spatial perception, we expect audio‐tactile integration in spatial tasks to occur late in development after the age of 12.

Yet, one piece of the puzzle needs to be included to complete the picture. Indeed, there is an ongoing debate about the necessity of vision in early life to develop spatial skills. On the one hand, studies have shown that early blind individuals have similar or superior performance to sighted individuals in tasks requiring an anatomical reference to determine the spatial location of sounds (Lessard et al. 1998; Röder et al. 1999; Voss et al. 2004; Battal et al. 2020) due to a sensory reorganization leading to compensatory behavior (Merabet and Pascual‐Leone 2010; Kupers and Ptito 2014). On the other hand, the same group of people lacks precision when localizing sounds using an external reference frame (Finocchietti et al. 2015; Gori et al. 2014; Vercillo et al. 2017). Such deficiency occurs because the lack of vision in early life prevents the calibration of vision over the other senses, resulting in the remaining sensory modalities building a noisy and ambiguous spatial representation (Gori 2015; Eimer 2004). Moreover, it appears that early blindness influences audio‐tactile integration. It has been observed that when performing a task with audio‐tactile spatial displacement (i.e., ventriloquist phenomenon), sighted and late blind adults mislocate the auditory stimulus erroneously toward the tactile one. However, this effect is reduced in congenitally blind participants (Occelli et al. 2012). The reduction of this effect in congenitally blind people might suggest a lack of multisensory integration in spatial tasks (Occelli et al. 2013). This leads us to the second point of interest: investigating audio‐tactile integration cross‐sectionally in blind children. In parallel, we aim to explore whether the development of precision in touch and hearing follows a similar trajectory and whether vision has an impact on this development. This information could provide important insight into the development of multisensory integration in the absence of vision.

To sum up, the present study pursues two primary objectives: (1) determining the developmental stage at which audio‐tactile performance yields a reduction in perceptual uncertainty, as predicted by the maximum‐likelihood estimation model (MLE) and (2) unraveling the role of vision in the development of auditory and tactile localization, as well as their integration. We circumscribe these goals to spatial representation.

To address both goals, we used an audio‐tactile localization task where participants received auditory, tactile, or audio‐tactile stimulations (spatially congruent or incongruent) delivered to the forearm. The use of uni‐modal conditions allows us to investigate the developmental trajectories of precision in these two sensory modalities and test the predictions of bimodal behavior according to the MLE model. The inclusion of the bimodal conditions allows us to test whether or not there is a reduction in uncertainty (i.e., better precision) following bimodal stimulation compared to uni‐sensory stimulation and whether such a reduction occurs optimally. Moreover, by discriminating between congruent and incongruent bimodal conditions, we can gain information regarding possible sensory dominance, causing a perceptual bias toward one modality over the other in the presence of a spatial incongruence similar to the ventriloquist effect. We tested a cohort of sighted children and adolescents aged 7–15 years, including relevant ages already investigated in previous studies on the development of multisensory integration (Gori et al. 2012; Petrini et al. 2014; Negen et al. 2019). This should allow us to identify the time windows in which optimal integration starts to emerge. Moreover, we included blind children and adolescents aged 7–15. We compared their performance with that of sighted peers to explore potential differences in uni‐sensory development of localization skills and audio‐tactile integration across different age groups.

## 2 Materials and Methods

### 2.1 Participants

We recruited a cohort of 110 sighted children and adolescents aged 7–15 years from local schools in Genoa (Italy). From the database provided by the Italian Institute of Technology (Genoa, Italy), we tested a group of 11 sighted adults (age: 32.95 years; SD = 11.63) to establish a general reference for typical adult performance in the uni‐sensory conditions, ensuring that thresholds for auditory and tactile conditions were comparable in terms of precision (see Supporting Information—Results section). All sighted participants reported normal or corrected‐to‐normal vision and no history of neurological or other sensory‐motor deficits.

We also recruited 19 blind children and adolescents aged 7–18 years. Nevertheless, the two older blind adolescents (B16 and B17) were not included in the analysis to ensure an age‐grouping more consistent with the sighted group. We included the performance of these two participants only in the plots with a special symbol to show their performance. Blind individuals were recruited from Hospitals (IRCCS Fondazione Mondino and IRCCS Eugenio Medea) and one rehabilitation center (Istituto David Chiossone) in Italy. All blind children were born with visual impairment (see Table S1 for details in the Supporting Information), and the criteria used to define blindness were based on the standard established by the World Health Organization as a best‐corrected visual acuity worse than 1.3 LogMAR.

To assess the developmental effect, both sighted and blind participants were divided into three groups following a similar approach to previous studies (Gori et al. 2012; Petrini et al. 2014; Negen et al. 2019; Rohlf et al. 2020): group 8–9 years, group 10–11 years, and group 12–15 years (see Table S2 for details). Each group included children from the lower age‐range plus one day, to the other end of the range plus 364 days, for example, the 8–9 age range includes children who are between 8.00 and 9.99 years old. Grouping participants in this manner enabled us to investigate whether qualitative changes in optimal integration emerge within distinct age groups, as demonstrated in prior studies (Gori et al. 2008; Scheller et al. 2021; Nardini et al. 2008). Adult participants gave written informed consent before starting the experiment, while for children, we collected informed consent from the parents or legal guardians. The local health service ethics committees approved the study (multicentric protocol approved by the The Ethics Committee of ASL 3 Genova, The Ethics Committee of Pavia Area, Fondazione IRCCS Policlinico San Mattero Pavia, and the Ethics Committee of IRCCS Eugenio Medea, sezione Scientifica dell'Associazione “La nostra Famiglia”: Prot. IIT_UVIP_MySpace N. 268/2021, May 3, 2021) and followed the tenets of the Declaration of Helsinki.

### 2.2 Apparatus and Stimuli

**FIGURE 1:**
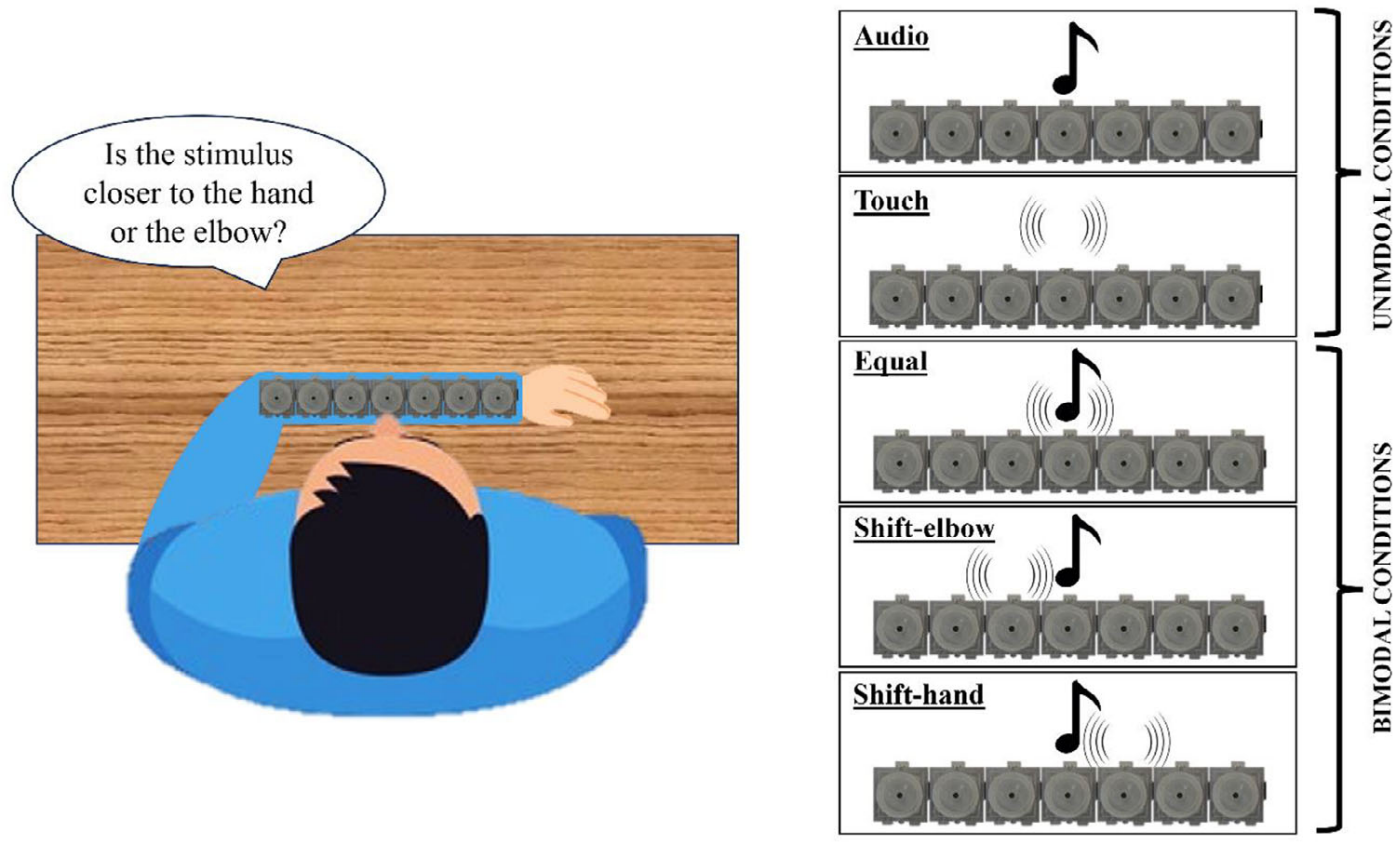
*Set up*—Each participant sat in front of a table with their heads facing straight. Once the device was attached to the forearm, the participant was asked to place the left arm parallel to the torso, finding a comfortable position so that the center of the device was aligned with the head. The participant's task was to judge whether the stimulus was closer to the hand or the elbow by pressing one of the two buttons of the mouse with the free hand. The task consisted of five randomly presented conditions: audio, touch, bimodal congruent (equal), in which the tactile and acoustic stimuli were presented in the same position, bimodal incongruent elbow (i.e., shift‐elbow: the tactile stimulus was shifted 9° in the direction of the elbow compared to the auditory stimulus), and bimodal incongruent hand (i.e., shift‐hand: the tactile stimulus was shifted 9° in the direction of the hand compared to the auditory stimulus).

The audio and vibrotactile stimuli were delivered by a customized device called MSI Caterpillar (Gori et al. 2019; Bollini et al. 2021) and controlled by Matlab 2018b. Each module was 3.5 cm large, covering ∼9° of visual angle. We used seven concatenated modules of this device to create a line and placed it on the left forearm of the participants (see Figure 1). The tactile stimuli were a 230 Hz vibration that oscillated continuously at that frequency for 10 ms duration, while the auditory stimuli were a 1 kHz beep lasting 50 ms. Stimuli could be audio‐only, touch‐only, or audio‐tactile and could come from one of the seven modules (i.e., probe's position: –27°, –18°, –9°, 0°, 9°, 18°, and 27°, where 0° represents the center of the device) decided adaptively by a QUEST. This adaptative algorithm uses a Bayesian approach to set the new position using all the information from previous trials (Watson and Pelli 1983).

The task consisted of five randomly presented conditions (Figure 1): audio, touch, bimodal equal (congruent stimuli), in which the tactile and acoustic stimuli were presented in the same position, bimodal incongruent in which there was a displacement between audio and tactile stimulation (shift‐elbow, with the tactile stimulus shifted 9° toward the elbow compared to the auditory stimulus, and shift‐hand, with the tactile stimulus shifted 9° toward the hand). Incongruent conditions were included to assess whether, at the bias level, one sensory modality dominated the final percept in a ventriloquist‐like fashion (Alais and Burr 2004; Gori et al. 2012; Rohlf et al. 2020). In previous studies investigating this in adults, an attraction of audio to the location of tactile stimulation was found (Caclin et al. 2002; Bruns and Röder 2010). Each condition was repeated 40 times for a total of 200 trials.

### 2.3 Procedure

Before starting the experiment, the sighted children had the opportunity to visually explore the device used to present the stimuli, while the blind children could explore it through touch. Participants sat in front of a table with their head facing straight ahead and looking at a point in front of them so that sighted children could not directly see the device during the task. The device was applied to the forearm as practicality to make the child's experience as minimally invasive as possible. In addition, the same device has already been used in the same location in previous studies (Gori et al. 2014; Vercillo and Gori 2015). After attaching the device to the forearm, the participant was asked to place the left arm parallel to the torso, finding a comfortable position so that the center of the device was aligned with the head (Figure 1). The participant's task was to determine whether the perceived stimulus was closer to the hand or the elbow by pressing one of the two buttons of the mouse with the free hand. During the session, the experimenter continuously checked that the child did not move their head and looked in front of them rather than at the device. The experiment lasted 15–20 min based on the child's needs.

### 2.4 Data Analysis

To analyze participants' precision at the group level, we fitted the probability of responding ‘comparison closer to the hand (i.e., right)’ with a generalized linear mixed model (GLMM) with a probit link function. The model included group (sighted, blind), condition (unimodal audio, unimodal touch, bimodal shift‐elbow, bimodal shift‐hand, bimodal‐equal), probe's position (–27°, –18°, –9°, 0°, 9°, 18°, 27°, with 0° representing the center of the device), and age group (8–9, 10–11, and 12–15) as fixed effects. All main effects, as well as all two‐way, three‐way, and the four‐way interactions among these factors were included. A random intercept for each participant was included to account for inter‐subject variability. Thus, the full model can be expressed as:

(1) $$\begin{array}{ccc} \Phi^{-1}(P\left(Hand_{i}\right) & = & \beta_{0}+\beta_{1}Position_{i}+\beta_{2}Condition_{i}\\ & & +\beta_{3}Group_{i}+\beta_{4}AgeGroup_{i}+\cdots \\ & & +\beta_{15}(Position_{i}\times \;Condition_{i}\times Group_{i}\\ & & \times \;AgeGroup_{i})+u_{0j} \end{array}$$

*Note*: The ellipsis represents all lower‐order two‐way and three‐way interactions among position, condition, group, and age group.

Where Φ^−1^ is the probit link function (i.e., the inverse function of the Cumulative Gaussian distribution), (*P*(*Hand_i_*) is the probability of “closer to the hand (i.e., right)” response in a trial *j*, β_0_ the intercept, β_*k*_ the fixed effect coefficients, and *u*_0*j*_ the random intercept for participant 𝑗.

To assess whether differences in precision exist across groups (sighted vs. blind) and age bins, we compared the full GLMM, including all interaction terms (Equation 1), against a reduced model containing only main effects and two‐way interactions (Equation 2) using a likelihood ratio test.

(2) $$\begin{array}{ccc} \Phi^{-1}(P\left(Hand_{i}\right) & = & \beta_{0}+\beta_{1}Position_{i}+\beta_{2}Condition_{i}\\ & & +\beta_{3}Group_{i}+\beta_{4}AgeGroup_{i}\\ & & +\beta_{5}\left(Position_{i}\times Condition_{i}\right)\\ & & +\beta_{6}\left(Position_{i}\times \;Group_{i}\right)\\ & & +\beta_{7}\left(Position_{i}\times \;AgeGroup_{i}\right)\\ & & +\beta_{8}\left(Condition_{i}\times \;Group_{i}\right)\\ & & +\;\beta_{9}\left(Condition_{i}\times \;AgeGroup_{i}\right)\\ & & +\beta_{10}\left(Group_{i}\times \;AgeGroup_{i}\right)+u_{0j} \end{array}$$

T‐statistics and corresponding *p* values for all fixed effects, including main effects and interactions, were derived directly from the GLME output and are integral to the model estimation. Therefore, no additional ANOVA or post hoc tests (i.e., no *F*‐statistics) were conducted, as the *t*‐values directly represent the inferential tests of the model's fixed effects. The model was implemented in MATLAB R2021a (MathWorks, MA, USA) using the *fitglme* function.

We also analyzed the data at the individual level to determine whether each participant integrated information optimally. We assessed each participant's performance by fitting psychometric functions to their data. For each condition, we fitted the proportion of “closer to the hand” responses plotted against the positions of the stimulus with a cumulative Gaussian distribution. From the psychometric functions, we extracted the inflection point, that is, the point of subjective equality (PSE), as a measure of accuracy, corresponding to the stimulus level in which the probability of “closer to the hand” response is 0.5, and the just noticeable differences (JND), as a measure of precision, that is calculated as the scaling parameter of the cumulative Gaussian distribution, directly affecting the width of the curve, corresponding to the stimulus values at which the probability of a given response (e.g., “closer to the hand”) is equal to 75% and 25%, respectively.

Following visual inspection of the psychometrics for each participant, we excluded ten sighted children (see Table S1 for details) due to inverted psychometric functions. Having an inverted psychometric is an indication that the child misunderstood the task, probably by reversing the response keys or simply not paying attention to the task. No blind participants were excluded.

We ran a 2 × 2 × 3 Repeated Measured ANOVA on the JNDs with the factors age‐group (8–9, 10–11, 12–15), group (sighted, blind), and bimodal condition (shift‐hand, shift‐elbow, equal). Since neither the main effect nor any of these interactions were significant, we averaged the JNDs across the three bimodal conditions. Thus, in subsequent analyses, the term *bimodal* refers to this average.

To evaluate whether participants benefit from the multisensory presentation, we first compared the bimodal JND to each participant's best unimodal JND (i.e., the lower of auditory or tactile). Comparing the performance in the bimodal condition to the auditory and tactile conditions individually might not reveal a significant increase in precision resulting from integration due to the risk of false positives, especially when there is a substantial difference in precision between the uni‐modals performance (Scheller and Nardini 2024). For this reason, instead of comparing the bimodal JNDs with each of the uni‐modal JNDs, we selected the best (i.e., lower) uni‐modal cue (JND) for each participant (Senna et al. 2021). We then compared it with the bimodal performance using a paired two‐tailed *t*‐test for each age‐group and group of participants—a necessary condition for integration.

As a second step, to assess if participants optimally integrated bimodal information, we calculated the predicted optimal bimodal performance for each of them based on the Maximum Likelihood Model (MLE) (Alais and Burr 2004; Ernst and Banks 2002). The MLE assumes that the optimal audio‐tactile precision is given by:

(3) $${\mathrm{JND}}_{\mathrm{optimal}}=\sqrt{\frac{{\mathrm{JND}}_{A}^{2}\;{\mathrm{JND}}_{T}^{2}}{{\mathrm{JND}}_{A}^{2}+{\;\mathrm{JND}}_{T}^{2}}}\;$$

where JND*_A_* and JND*_T_* are the audio and tactile unimodal JNDs, the improvement is greater when σ*_A_*_ = _σ*_T_*.

Following Equation (3), the MLE‐predicted optimal JND was tested against the JND obtained from the bimodal psychometric curves for each participant. Notably, the MLE prediction assumes that the optimal combination of multiple sensory cues determines an increase in precision (and, consequently, a reduction of the JND) in the bimodal compared to the unimodal conditions. Thereby, if two stimuli from different sensory modalities are combined according to the MLE model, the bimodal JND will be similar to the MLE prediction. We tested this hypothesis by comparing bimodal JND with the prediction obtained from the MLE for each age‐group and group of participants using a paired two‐tailed *t*‐test.

To ensure the robustness of the results, all the *t*‐test analyses are reported with the same analysis using Bayesian statistics, reporting the Bayesian factor (BF_10_). For both the ANOVA and the *t*‐tests, a BF less than or equal to 0.33 suggests that the evidence supports the null hypothesis over the alternative hypothesis. A BF greater than 3 indicates evidence in favor of the alternative hypothesis.

## 3 Results

The GLMM revealed differences in precision across groups (sighted and blind) and age bins. Indeed, the likelihood ratio test comparing the full model (including three‐ and four‐way interactions) with a reduced model (including only main effects and two‐way interactions) revealed that the full model significantly outperformed the reduced model, χ2(4) = 456.12, *p* < 0.001. This indicates that the higher‐order interactions among condition, group, age bin, and position significantly enhances the model's explanatory power and should be retained in the model. These interactions suggest that the effect of condition on participants’ responses varies depending on group membership and age, reflecting differences in precision across sighted and blind participants and across age bins. Overall, the GLMM showed that the group of blind participants is more precise compared to the sighted counterpart. Nevertheless, we can summarize the results in three main findings: bimodal integration starts to develop between 12 and 15 years of age in sighted participants but not in blind children; auditory and tactile localization shows a different developmental trend, and this trend differs between the two groups. The descriptive statistics (means and standard deviations) for all groups and conditions are summarized in Table 1. For simplicity, results in the bimodal and unimodal conditions will be discussed in two separate paragraphs.

**TABLE 1:** Summary of descriptive statistics (mean ± SD) for all experimental groups and conditions.

	BLIND groups				SIGHTED groups	
	8–9 years	JND	SEM	8–9 years	JND	SEM
Conditions	Audio	8.9671	1.5652	Audio	29.6531	4.6694
	Touch	8.5446	2.14985	Touch	15.6767	1.99595
	Shift‐elbow	8.3172	1.9252	Shift‐elbow	13.8113	1.5612
	Shift‐hand	7.8601	1.7312	Shift‐hand	13.4085	1.4427
Equal	9.8002	2.6758	Equal	18.0297	2.4902

	10–11 years			10–11 years		
Conditions	Audio	7.6735	1.0094	Audio	14.7172	1.01465
	Touch	7.8213	1.4747	Touch	9.7667	0.69235
	Shift‐elbow	6.2527	1.0872	Shift‐elbow	8.6484	0.5895
	Shift‐hand	6.4169	1.0509	Shift‐hand	7.995	0.4922
Equal	6.2315	1.0117	Equal	9.6836	0.6877

	12–15 years			12–15years		
Conditions	Audio	9.1325	1.0834	Audio	11.1767	0.73925
	Touch	5.0419	0.59445	Touch	8.5862	0.6453
	Shift‐elbow	5.8405	0.7365	Shift‐elbow	6.8742	0.4535
	Shift‐hand	5.0405	0.5793	Shift‐hand	6.6452	0.4181
	Equal	7.0534	0.9399	Equal	7.4982	0.5161

### 3.1 Bimodal Integration Results

The GLMM results comparing blind and sighted participants across the bimodal conditions show that blind participants are more precise than their sighted peers at a younger age. As age increases, the precision of the sighted participants improves, gradually approaching that of the blind participants, especially for the bimodal‐equal condition. In fact, while the two groups significantly differ at age 8–9 in all bimodal conditions (bimodal‐equal: *t* = 2.45, *p* = 0.014; shift‐hand: *t* = 2.60, *p* = 0.009; shift‐elbow: *t* = 2.41, *p* = 0.016), at age 10–11 they still differ only in the bimodal‐equal condition (*t* = 2.66, *p* = 0.008), but not in the shift‐hand (*t* = 1.48, *p* = 0.14) and in the shift‐elbow condition (*t* = 1.90, *p* = 0.057). At age 12, the two groups do not differ in the equal condition (*t* = 0.53, *p* = 0.60) and in the shift‐elbow condition (*t* = 1.35, *p* = 0.18), while the sighted are less precise in the shift‐hand condition (*t* = 2.34, *p* = 0.019).

**FIGURE 2:**
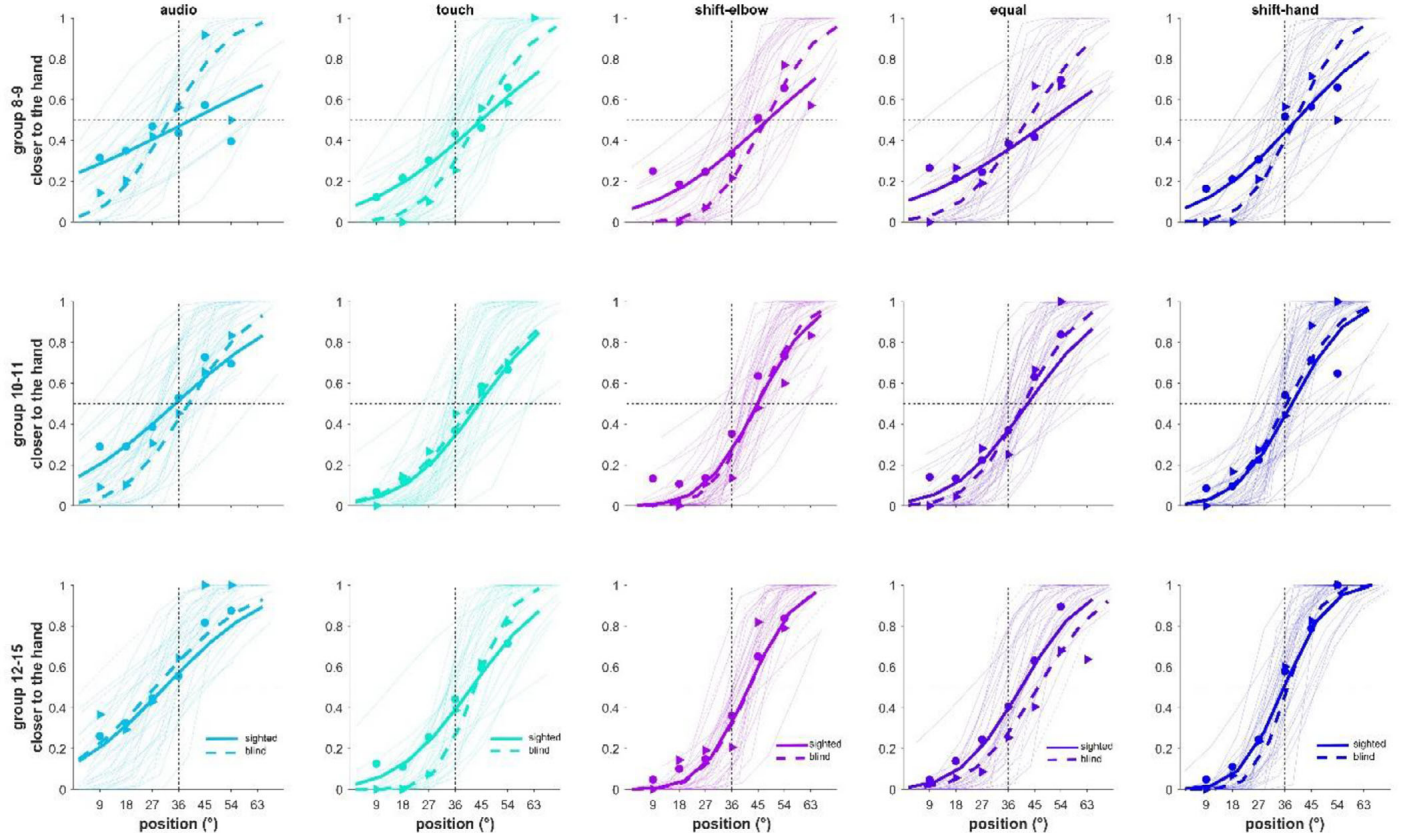
Results of psychometric functions for the sighted (solid line) and blind (dashed line) groups divided by conditions and age groups. The thin fits represent the psychometric fits for each participant, while the thick lines are the super‐subject fits.

Regarding the analyses performed at the individual level, Figure 2 shows the psychometric functions plotting both the results of a super‐subject (thicker lines and symbols) and individual psychometrics (thinner lines) for each condition (columns), age‐group (rows) and groups (solid lines sighted and dashed lines blinds).

**FIGURE 3:**
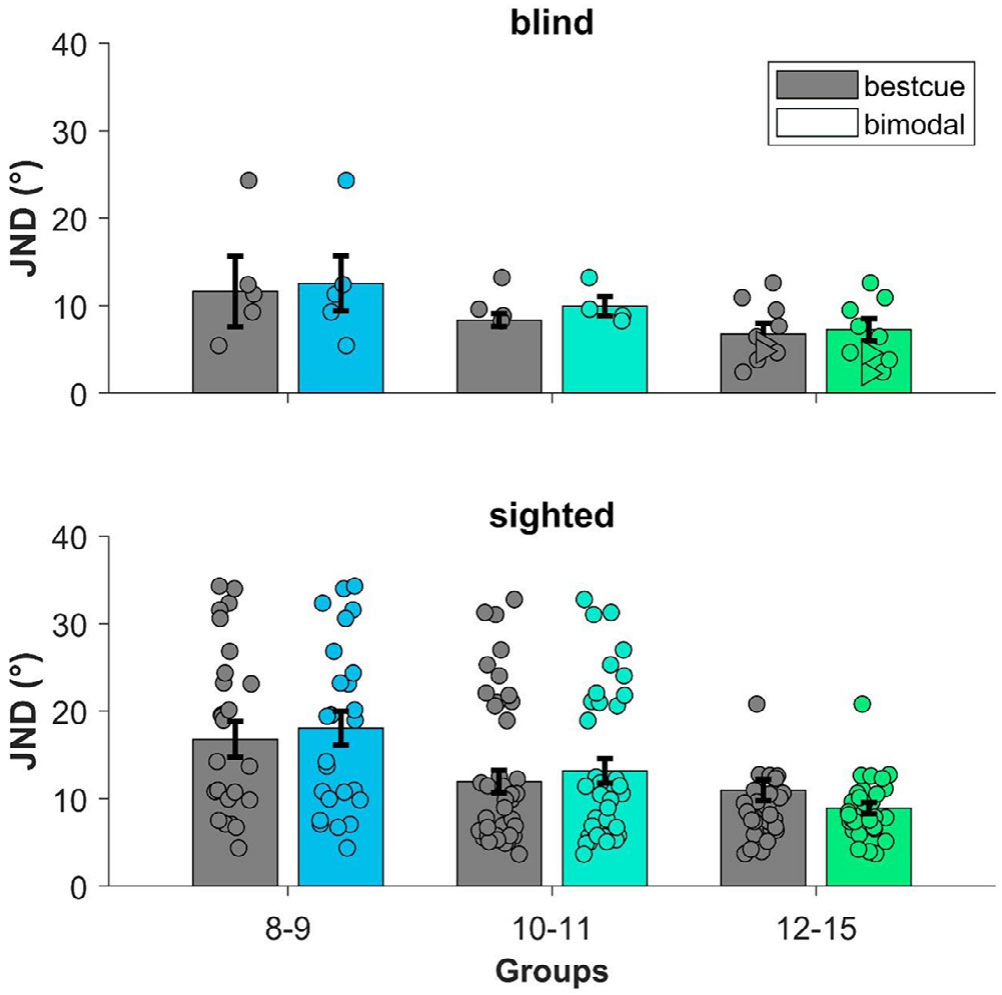
The bar plots represent the average JNDs of the bestcue (in gray), selected from uni‐modal data and the average bimodal JNDs (colored) for each age‐group and participant group. Circle symbols represent the data points for each participant. Triangle symbols in the top right part of the plot are the data of the “older” blind participants excluded from the statistical analysis. Error bars represent ± s.e.m.

The results of bimodal JNDs and best unimodal JNDs (best cue) for the blind group are reported in Figure 3, top row. For none of the age groups did we find a significant difference between the best unimodal cue and *bimodal* (8–9: t_4_ = 0.599, *p* = 0.58, *d* = 0.27, BF_10_ = 0.46; 10–11: t_3_ =  1.37, *p* = 0.26, *d* = 0.68, BF_10_ = 0.78; 12–15: t_7_ = 0.46, *p* = 0.66, *d* = 0.16, BF_10_ = 0.37). In the sighted group, we found the same result for the age groups 8–9 (t_24_ = 0.67, *p* = 0.51, *d* = 0.13, BF_10_ = 0.26) and 10–11 (t_37_ = 1.24, *p* = 0.22, *d* = 0.2, BF_10_ = 0.35). In contrast, for the group of children aged 12–15, the precision is higher (i.e., smaller JND) in the bimodal condition than in the best unimodal cue (t_28_ = 2.55, *p* = 0.016, *d* = 0.47, BF_10_ = 3.01), in line with the expectation that optimal integration of bimodal cues should result in enhanced precision compared to unimodal cues.

**FIGURE 4:**
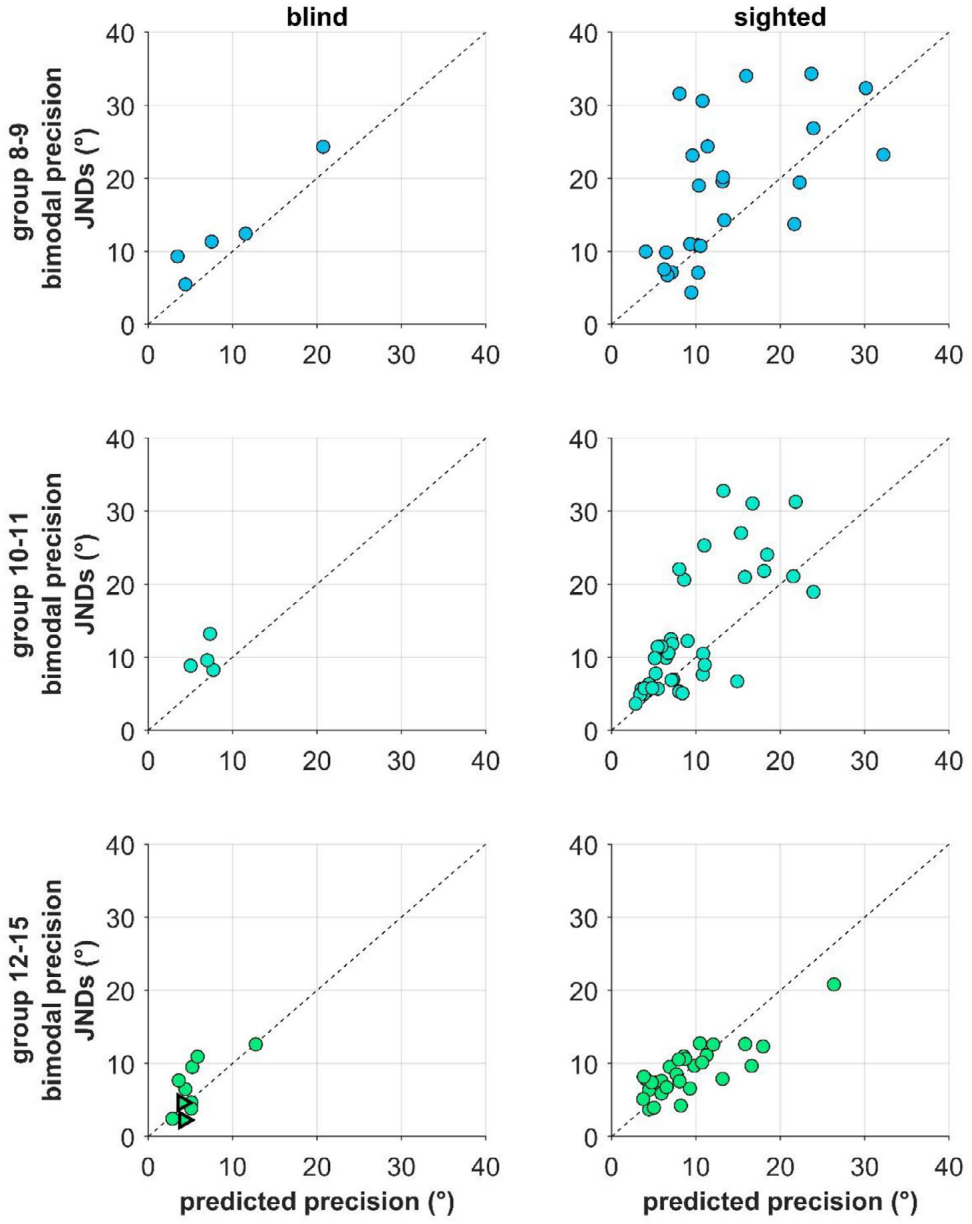
Individual data of precision (i.e., JNDs) plotted against the precision predicted by the MLE model for each age group and bimodal condition. On the left column are reported the data of the blind group, while sighted data are in the right column. The dashed lines represent equality lines. Values above this line indicate that bimodal precision is lower than predicted precision. The triangle symbols in the bottom left plot are the data of the “older” blind participants excluded from the statistical analysis.

These results are supported by the comparison between the observed bimodal precision and the precision predicted by the MLE at the individual level. Results for both groups are presented in Figure 4. As might be expected for both groups in the 8–9 age group (blind: t_4_ = 3.24, *p* = 0.03, *d* = 1.45, BF_10_ = 3.1; sighted: t_24_ = 2.68, *p* = 0.013, *d* = 0.54, BF_10_ = 3.83), the bimodal JNDs are significantly larger than the MLE prediction, as most of the data lies above the equality line (dashed line). The same result is also clear for the sighted group at 10–11 (t_37_ = 3.74, *p* < 0.001, *d* = 0.6, BF_10_ = 47.97). In contrast, it is clear that there is no significant difference between observed and predicted bimodal performance for the sighted group at 12–15 (t_28_ = 0.34, *p* = 0.74, *d* = 0.06, BF_10_ = 0.21), suggesting that at this age, sighted children integrate sensory information optimally. It is important to note that for the group of blind children aged 10–11 and 12–15, the results are anecdotal, not allowing for a definitive conclusion. The bimodal JNDs do not turn out to be significantly different from the MLE prediction, although with decreasing age, this nondifference tapers off (10–11: t_3_ = 2,88, *p* = 0.06, *d* = 1.44, BF_10_ = 2.02; 12–15: t_7_ = 1.83, *p* = 0.11, *d* = 0.65, BF_10_ = 1.07). However, we believe that this lack of significance is mainly attributable to the small sample size in both age groups and interindividual variability, rather than providing evidence of optimal sensory integration. Thus, this current finding does not permit a definitive conclusion regarding the integration strategy. The lack of significant difference from either the best unimodal cue or the MLE prediction suggests that the task may not have been sensitive enough to detect potential multisensory benefits, even if present. Future studies with a larger sample and/or a slightly different task may help to clarify the developmental trajectory of multisensory integration in this context.

### 3.2 Unimodal Results

**FIGURE 5:**
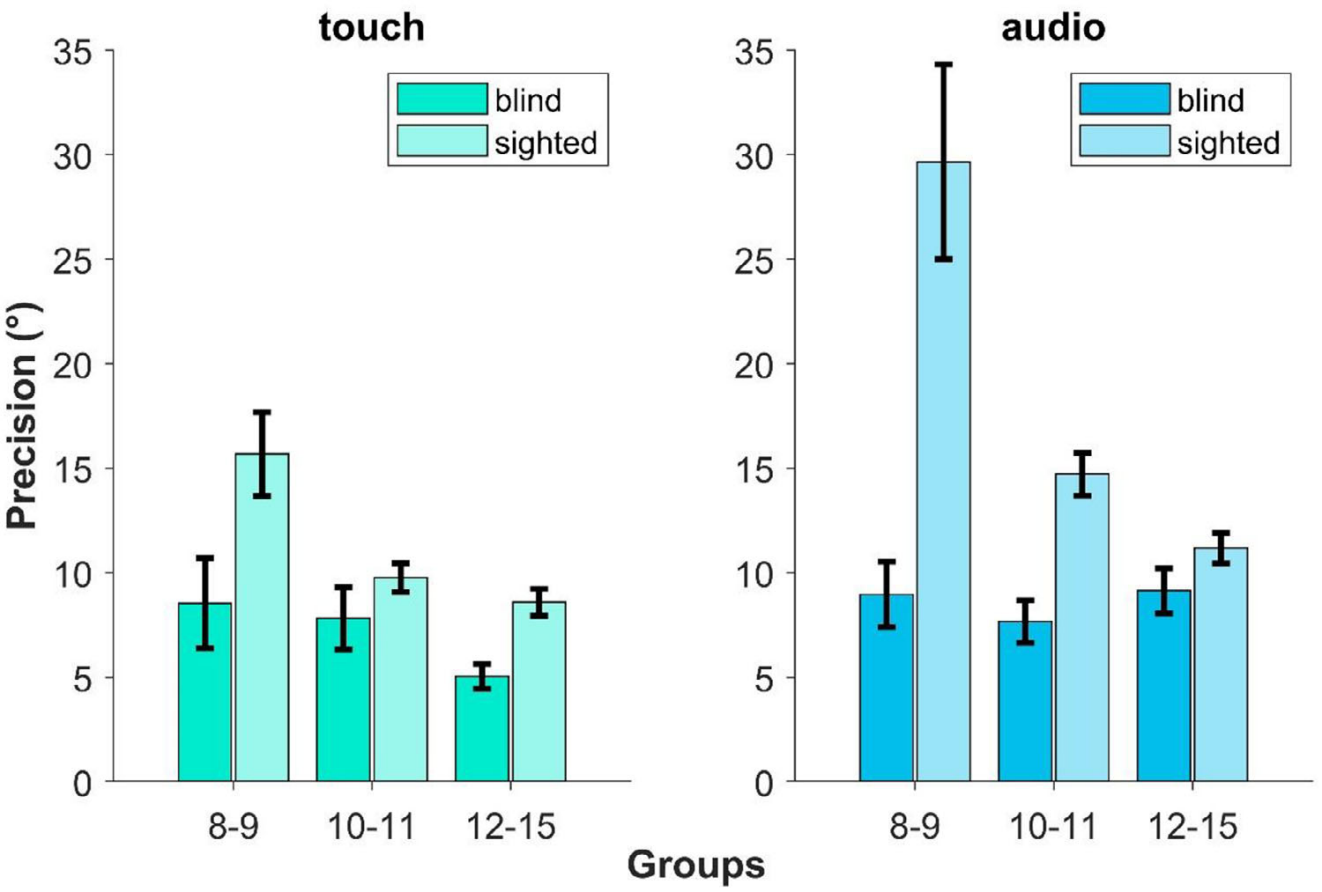
Bar plots representing the average precision of the tactile (on the left) and auditory (on the right) precision obtained from the GLMM for both groups—blind (darker bars) and sighted (lighter bars)—for each age group. JNDs are estimated at the 75th percentile, and error bars represent ± s.e.m.

In the sighted group, auditory localization (Figure 5 on the right) is systematically less precise than tactile localization (Figure 5 on the left). As shown by the GLMM output, audio differs from touch in each age group (8–9: *t* = 3.76, *p* < 0.001; 10–11: *t* = 4.77, *p* < 0.0001; 12–15: *t* = 3.1, *p* = 0.002). Moreover, if we compare the different age groups within each modality, we observe that the precision in the tactile modality begins to stabilize earlier than in the auditory modality. Indeed, in the tactile modality, precision stops improving between the ages of 10 and 11 (8–9 vs. 10–11 years old: *t* = 4.58, *p* < 0.0001; 10–11 vs. 12–15: *t* = 1.59, *p* = 0.11). In contrast, precision in auditory localization continues to improve beyond age 12 (8–9 vs. 10–11: *t* = 4.91, *p* < 0.0001; 10–11 vs. 12–15: *t* = 2.86, *p* = 0.004). These results in sighted participants show different developmental trajectories for the two sensory modalities in spatial tasks. The tactile modality stabilizes around age 10–11, whereas auditory performance continues to improve until the oldest age group, most likely because there is more room for improvement since auditory precision is worse than the tactile one.

The results of the GLMM for the blind group show a different pattern. The precision in auditory localization seems to be refined earlier than in touch. There is no different precision for audio across groups (8–9 vs. 10–11: *t* = 0.74, *p* = 0.46; 8–9 vs. 12–15: *t* = 0.09, *p* = 0.93; 10–11 vs. 12–15: *t* = 0.98, *p* = 0.32). In contrast, for touch, precision stabilizes later at around 12 years of age (10–11 vs. 8–9: *t* = 0.4, *p* = 0.69; 8–9 vs. 12–15: *t* = 2.88, *p* = 0.004; 10–11 vs. 12–15: *t* = 2.68, *p* = 0.007). This pattern is precisely the opposite of what happens in the sighted group, where, as we aforementioned, tactile localization develops first, and only after does auditory localization. Moreover, while in sighted participants, auditory localization is significantly worse than touch already in the youngest group, this is not the case for the blind participants in which there is no difference between audio and touch, even in the younger groups (8–9 group: *t* = 0.19, *p* = 0.84; 10–11 group: *t* = 0.11, *p* = 0.92). Only in the age‐group 12–15, there is a boost of tactile localization, which became more precise than audio (*t* = 3.85, *p* = 0.0001).

The GLMM results comparing blind and sighted participants reveal that blind participants are more precise in auditory localization compared to the sighted peers already at 8–9 years (blind vs. sighted, 8–9: *t* = 3.97, *p* < 0.0001; 10–11: *t* = 3.57, *p* = 0.0004), but not in the oldest group tested (12–15: *t* = 1.42, p = 0.16). Similarly, for tactile localization, blind participants are more precise than their sighted peers already at 8–9 years (*t* = 2.5, *p* = 0.012). However, once participants reach the age of 10–11, this difference disappears (*t* = 1.38, *p* = 0.17), most likely because tactile localization in the blind group remains constant between 8 and 11, while in the sighted group, there is an improvement. Instead, in the 12–15 age‐group, there is again a difference between the two groups (*t* = 3.96, *p* < 0.001) due to an increase in precision in the tactile condition in the blind group.

## 4 Discussion

In the present study, we investigated the development of localization skills using both auditory and tactile inputs, as well as the benefits of multisensory processing. We tested a cohort of children and adolescents aged 7–15 years to identify the developmental time course of optimal integration. Moreover, we explored the contribution of early visual experience to such development by comparing the performance of blind children with their sighted peers.

The present study yields three main findings. The most relevant finding concerns the development of optimal multisensory integration, showing that, in spatial tasks, this development is influenced by both age and visual experience. The other two results concern the development of uni‐sensory localization. First, we found that the two sensory modalities tested, audition and touch, follow two different developmental trajectories. Second, this developmental trend differs between sighted and blind children.

### 4.1 Development of Audio‐Tactile Localization and the Influence of Visual Experience

In this study, we found evidence of optimal audio‐tactile integration only in the oldest group of sighted children. We did not observe a significant multisensory benefit in blind children, and Bayesian analysis confirms the absence of such an effect. However, comparison with the optimal prediction yielded inconclusive results, suggesting the need for further investigation with larger samples or more sensitive tasks to clarify the integration strategy in this group. According to the “optimal cue integration theory” (Ernst and Bülthoff 2004; Ernst and Banks 2002), the brain adeptly merges sensory cues to enhance perceptual precision. The brain reduces ambiguity by strategically combining sensory information through the maximum‐likelihood estimation model (MLE), improving overall perceptual acuity by reducing noise in the estimate and fostering a more dependable representation of the external environment (Ernst and Bülthoff 2004; Ernst and Banks 2002). Nevertheless, optimal cue integration is not inherently present at birth. During childhood, the optimal combination is traded with complete reliance on the most precise sense specific to the perceptual domain. For example, in a spatial localization task, vision—the dominant sense for space (Alais and Burr 2004)—completely overtakes perception, guiding the calibration of other modalities and allowing adjustments following physical growth (Burr and Gori 2012; Ernst 2008). In the present experiment, we demonstrated that this late onset of optimal multisensory integration is not exclusive to scenarios involving the dominant sensory modality for the task (i.e., vision), but also extends to include bimodal perception resulting from the combination of non‐dominant senses. We found that audio‐tactile optimal integration in a spatial localization task occurs around the age of 12, similar to what happens for visuo‐auditory spatial tasks (Gori et al. 2012) and other perceptual domains involving audio‐tactile integration (Scheller et al. 2021; Petrini et al. 2014; Stanley et al. 2023). Nevertheless, in blind children, we did not find a significant audio‐tactile benefit compared to uni‐sensory presentation in line with previous research suggesting a lack of multisensory integration in spatial tasks (Occelli et al. 2012; Occelli et al. 2013). However, it is essential to consider that in the present study, blind children demonstrated greater precision than their sighted peers in the localization of uni‐sensory and multisensory stimuli, regardless of age. Therefore, we should be cautious in concluding that blind children do not show optimal integration. Instead, it is possible that, given that blind individuals already exhibit high precision in performing the localization task in all the conditions tested, there is little to no room for measurable improvement when multisensory stimuli are introduced in this specific task. This is particularly evident in the oldest blind group, where the results in the bimodal cues do not differ from either the best cue or the MLE prediction. Bayesian analyses for these comparisons yielded Bayes factors in the range of anecdotal evidence for the null hypothesis, supporting a cautious interpretation of the results regarding the absence of optimal integration. As an additional check, we calculated the signal‐to‐noise ratio for each participant, which captures the relative reliability of the two uni‐sensory inputs. Although high ratios (e.g., >2) are generally associated with limited integration benefits, our data showed that signal ratios were well balanced across groups and ages. This suggests that the lack of statistically significant multisensory enhancement, particularly in blind children, is unlikely to be due solely to an imbalance in signal reliability. We therefore interpret these data with caution, noting the need for more sensitive paradigms or larger samples to draw stronger conclusions for this age group. We therefore interpret these data with caution, acknowledging the need for more sensitive paradigms or larger samples to draw firm conclusions for this age group. It is possible that making the task more difficult, perhaps with higher spatial resolution, might leave room for multisensory improvement. Indeed, based on the inverse efficiency principle of multisensory integration (Stein and Meredith 1993), the higher the reliability of uni‐sensory inputs, the lower the likelihood of multisensory interaction. Moreover, as mentioned above, sensory information is integrated to minimize sensory uncertainty and produce an overall perceptual improvement (Ernst and Bülthoff 2004). However. this principle may not apply to the group of blind people who are already extremely precise in this task.

In other perceptual domains, such as size perception (Scheller et al. 2021), it has been shown that blind children do optimally integrate audio‐tactile information. This different result could be due to the fact that vision is not essential in the development of size perception. In fact, even in sighted participants, touch is the dominant sensory modality for size perception, calibrating the other senses in the task (Gori et al. 2008), and blindness early in life does not impact audio‐tactile integration (Scheller et al. 2021). In contrast, vision is the dominant sense in spatial tasks (Alais and Burr 2004; Gori et al. 2014), regardless of the other sensory modalities tested. Indeed, as aforementioned, the lack of visual experience activates cross‐modal compensation mechanisms, boosting superior spatial abilities in both tactile (Goldreich and Kanics 2003; Wong et al. 2011) and auditory (Battal et al. 2020; Voss et al. 2015) localization that rely on anatomical coordinates.

Our experiment also provides insights into perceptual accuracy (see Supporting Information, Section “Perceptual biases: ventriloquist effect”). In the shift‐hand and shift‐elbow conditions, where there is a spatial conflict between the position of the auditory and tactile stimulus, perception has a bias toward the actual position of the tactile stimulus (ventriloquist‐like effect). However, this effect seems to be reduced in the older group 12–15, in which the bias seems to lie somewhere between the real positions of the auditory and tactile stimuli. Also, note that across all groups, there seems to be no difference between the two blind and sighted groups. However, these observations are currently only descriptive and further studies are needed to investigate this aspect.

### 4.2 Development of Auditory and Tactile Localization

Additionally, through this study, we could define and directly compare the developmental trajectories of auditory and tactile localization abilities in sighted and blind children.

In sighted children, it has been previously demonstrated that the ability to discriminate both tactile (Bremner et al. 2008) and acoustic inputs are already present in infants as young as 5–6 months (Muir et al. 1989) and continues to develop with age (Bremner and Spence 2017; Kühnle et al. 2013). In this study, we observed that the refinement process of localization ability for tactile information tends to stabilize around 10–11 years, whereas auditory localization shows potential for improvement beyond 12 years of age. Moreover, we found that sighted children have better tactile than auditory localization precision. This may be attributed to the greater importance of localizing tactile stimuli on the body than auditory stimuli, as they may represent potential threats. Moreover, tactile spatial perception primarily emerges from a continuous exchange of feedback among tactile, proprioceptive, and visual information regarding the position of the limbs and the object touching the skin (Heed et al. 2015), which probably enables it to reach adult‐like precision levels earlier in development than auditory spatial perception.

In blind children, we found that the developmental pattern of the two sensory modalities differs from that of their sighted peers. While auditory precision does not vary within the tested age range for blind participants, we observe a marked improvement in tactile precision at ages 12–15.

Moreover, when we compared the performance of blind and sighted peers, we found that blind children are more precise already at around 8 years of age (the lowest age tested) than their sighted peers for both modalities. These results are consistent with previous studies on blind adults, which demonstrated superior spatial abilities in both tactile (Goldreich and Kanics 2003; Wong et al. 2011) and auditory (Battal et al. 2020; Voss et al. 2015) localization compared to sighted individuals. This is because blind adults utilize anatomical (or somatotopic) coordinates of space to localize single inputs independently from the nature of the spatial task and do not exhibit phenomena of spatial remapping present in sighted individuals, such as the cross‐hand effect (Gori et al. 2014; Röder et al. 2004; Bollini et al. 2023). The inherent structure of the experimental paradigm used may have amplified this result. Indeed, in the present task, localization was strongly connected to body coordinates, as the evaluation of the stimulus position was anchored to the arm (closer to the elbow or the hand).

### 4.3 Limitations of the Study

The present study has limitations that may restrict the generalizability of the results. The main limitation is the sample of blind participants. Although valuable, its size reduces the statistical power to detect strong developmental trends or the effects of multisensory integration. Additionally, the age distribution within the blind group may introduce variability, as developmental differences might not be evenly represented. Another potential limitation is the task design, which heavily relies on body‐centered reference frames. This might limit the exploration of how spatial skills develop in external reference frames, especially in blind individuals, where such frames are often less precise (Finocchietti et al. 2015; Gori et al. 2014). Future studies should aim to include larger age groups and explore tasks with varying degrees of spatial complexity to better understand multisensory integration across different sensory and spatial contexts.

## 5 Conclusion

We can conclude that optimal multisensory integration occurs late in development in tasks involving explicit judgments about the localization of stimuli in space, even when vision is not directly involved in the task. In our case, the integration of audio‐tactile localization follows the same developmental stages of audio‐visual localization (Gori et al. 2012). In sighted children, optimal integration emerged only in the oldest age group (12–15 years), whereas blind children did not show a measurable audio‐tactile benefit in any of the tested age groups. In the blind group aged 12–15, bimodal precision did not differ significantly from the best unimodal cue, and Bayesian analyses (BF = 0.37) support the absence of multisensory benefit. In contrast, the comparison between bimodal accuracy and optimal prediction yielded inconclusive results (BF = 1.07), suggesting that the current task may not have the sensitivity or statistical power necessary to detect integration, even if present. It is important to note that increasing the sample size could clarify this point, but the result could go either way. When vision is absent in the developmental process, sensory compensation is activated, leading to an enhancement of other sensory modalities to the extent that the benefit produced by multisensory inputs becomes redundant. However, we believe that this effect is closely related to the spatial reference frame involving anatomical coordinates, and increasing the degree of difficulty of the task might bring out an integration effect even in the blind.

## Funding

This research was funded by the MYSpace project and the European Research Council (Grant g.a. No 948349) to Monica Gori and in part by Ministero della Salute, Grant RC2025‐2026 to Francesca Tinelli.

## Conflicts of Interest

The authors declare no conflicts of interest.

## Supporting information




**Supporting File 1**: desc70094‐sup‐0001‐SuppMat.docx

## Data Availability

The authors have nothing to report.
