# Supplementary material for: The Development of Audio‐Tactile Spatial Integration: Unraveling Vision's Contribution
Source: Dev Sci. 2025 Nov 14;29(1):e70094. doi: 10.1111/desc.70094 (PMC12617394; doi:10.1111/desc.70094)
Supplement: Supplementary file 1 — Supporting File 1: desc70094‐sup‐0001‐SuppMat.docx [file DESC-29-e70094-s001.docx]

**Supplementary Materials**

**The development of audio-tactile spatial integration: unraveling vision's contribution.**

*Alessia Tonelli ^1,2 *^, Irene Senna*^3¶^*, Maria Bianca Amadeo^1^*^¶^*, Walter Setti^1^, Nicola Domenici^1^, Sabrina Signorini^4^, Elena Cocchi^5^, Giuseppina Giammari^6^, Sandra Strazzer^6^, Francesca Tinelli^7^, Paola Camicione^8^, Massimiliano Serafino ^8^, Monica Gori^1^*

^1^Unit for Visually Impaired People (U-VIP), Istituto Italiano di Tecnologia, Genoa, Italy

^2^ Schoool of Psychology, The University of Sydney, Sydney, Australia

^3^Department of Psychology, Liverpool Hope University, Liverpool, UK

^4^Developmental Neurophthalmology Unit, IRCCS Mondino Foundation, Pavia, Italy

^5^Fondazione David Chiossone, Genova, Italy

^6^ Scientific Institute, IRCCS E. Medea, Bosisio Parini, Lecco, Italy

^7^Department of Developmental Neuroscience, IRCCS Fondazione Stella Maris, Pisa, Italy

^8^Istituto Gianna Gaslini, Genova, Italy

**Materials.**

*Participants*

Below is the table with the clinical information of the blind children and adolescents tested.

Table S1. Clinical details of the blind group

| **Participant** | **Sex** | **Age at  the testing** | **Diagnosis** | **Visual Residue** |
| --- | --- | --- | --- | --- |
| B01 | m | 13.58 | inherited retinal dystrophy | 0,5/10 |
| B02 | m | 9.5 | inherited retinal dystrophy | light perception |
| B03 | f | 9.58 | inherited retinal dystrophy | light perception |
| B04 | m | 9.67 | inherited retinal dystrophy | light perception |
| B05 | m | 7.58 | congenital cataract (bilateral) | 0,5/10 |
| B06 | f | 10.08 | congenital glaucoma (bilateral) | 0,5/10 |
| B07 | m | 12.25 | Encephalocele | light perception |
| B08 | f | 11.25 | Leber Amaurosis | light perception |
| B09 | f | 10.67 | Leber Amaurosis | no vision |
| B10 | f | 11.42 | microphthalmia  and anophthalmia | no vision |
| B11 | f | 12.58 | congenital glaucoma  (bilateral) | light perception |
| B12 | f | 15.17 | Neurofibromatosis | light perception |
| B13 | m | 16.33 | retinablastoma | no vision |
| B14 | f | 15.33 | disp.setto-ottica | no vision |
| B15 | m | 12.5 | Retinopathy | light perception |
| B16 | f | 18.12 | optic nerve hypoplasia (bilateral) | light perception |
| B17 | f | 17.33 | Leber Amaurosis | light perception |
| B18 | f | 6.5 | ocular albinism | light perception |
| B19 | m | 13.83 | Retinopathy | light perception |

| ***Groups*** | ***N. of children tested*** | ***Average Age*** | ***Standard Deviation*** | ***N. of children excluded*** | ***N. of children analyzed*** |
| --- | --- | --- | --- | --- | --- |
| 8-9 sighted group | 33 | 9.1 | 0.74 | 8 | 25 |
| 10-11 sighted group | 45 | 11.08 | 0.55 | 7 | 38 |
| 12-15 sighted group | 32 | 12.72 | 0.86 | 3 | 29 |
| 8-9 blind group | 5 | 8.57 | 1.44 | 0 | 5 |
| 10-11 blind group | 4 | 10.85 | 0.61 | 0 | 4 |
| 12-15 blind group | 10 | 14.65 | 1.99 | 2 | 8 |

Table S2. The age range of sighted and blind children divided into three groups: group 8-9, group 10-11, and group 12-15. The age range includes children from the age of one year plus one day to one year plus 364 days, e.g., the 8-9 age range includes children who are between 8.00 years old to 9.99 years old.

*Additional Data Analysis*

To assess whether differences in uni-sensory cue reliability may have limited the potential for measurable multisensory benefits, we computed the cue noise ratio for each participant, defined as:

$$cue ratio=\frac{max\left( {JND}_{audio}, {JND}_{touch} \right)}{min\left( {JND}_{audio}, {JND}_{touch} \right)}$$

This metric captures the relative reliability of the two sensory cues, with higher values indicating a greater imbalance. Recent theoretical and empirical work suggests that when cue ratios exceed a value of ~2, the improvement from multisensory integration becomes minimal and may not be detectable in behavioral performance ^1,2^. However, no inferential statistical comparisons were conducted against a specific value.

In a second additional analysis in the multisensory conditions, we calculated the shift index to quantify the influence of one sensory modality on the other regarding accuracy in performing the localization task. To calculate this index, we normalized the PSEs of the incongruent elbow (shift-elbow) and incongruent hand (shift-hand) conditions by subtracting the PSE of the equal condition from each value. Positive values indicate that the perception is shifted toward the hand, while negative values indicate that the stimulus is perceived closer to the elbow.

**Results**.

*Stimuli Selection*

Before starting the experiment, the intensities of the tactile and auditory stimuli were adjusted to yield comparable reliabilities (i.e., JNDs) across the two unisensory modalities. This was validated in a pilot study with 11 adults. While this comparison may not fully generalize to children, it provided a foundation for selecting stimulus parameters, ensuring the unisensory modalities were approximately matched in precision before testing the child sample. There was no difference in the localization precision between the auditory (JND = 6.22°, SD = 2.028) and tactile (JND = 5.97°, SD = 1.71) JNDs, as shown by a GLMM with condition, probe’s position, and their interaction as fixed effect predictors, and the random intercept as random factor ( audio vs touch, t = 0.38, p = 0.70).

*Cue ratio analysis*

Descriptive results are reported in Figure 1S. Across age groups and participant groups, cue ratios were generally close or below 2, suggesting that auditory and tactile precision were relatively balanced for most participants. In both sighted and blind children, mean cue ratios ranged on average from 1.5 to 2 across age groups. These findings suggest that cue imbalances were not large enough to fully suppress the emergence of multisensory benefits and should therefore not be considered the main limiting factor in our results.

*
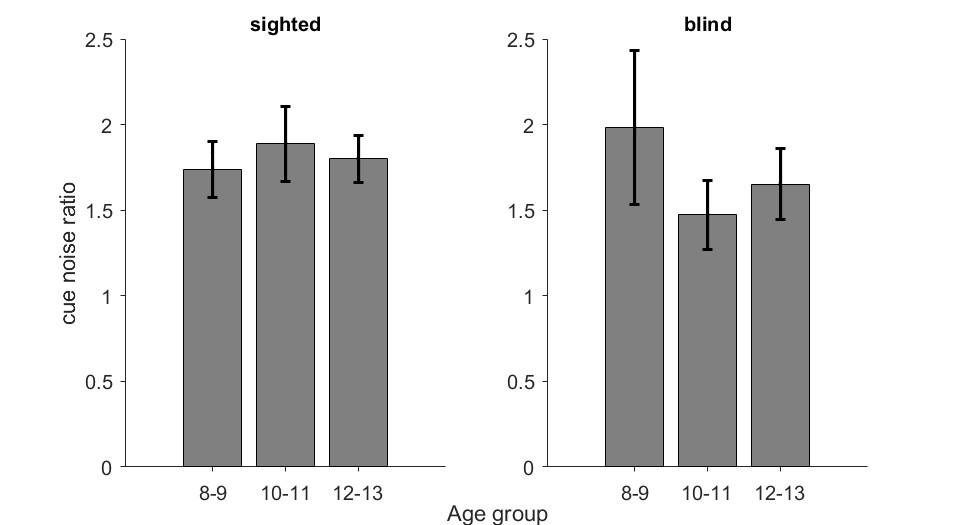
*

Figure 1S. Average cue noise ratios (worst/best unisensory JND) for all participants, grouped by age (8–9, 10–11, 12–15 years) and visual status (sighted, blind).

*Perceptual biases: ventriloquist effect*

Lastly, we also want to mention a qualitative evaluation of the accuracy results for the localization task represented as the PSEs of the psychometric functions minus the center of the array that corresponds to the position of the sound in bimodal conditions. In Fig. 2S, the average bias for each participant group is reported (darker colors sighted, lighter colors blind) subdivided per age group. The lines indicate the average bias in the uni-sensory conditions for blind (dashed line) and sighted (continuous line) participants. Moreover, with the black dotted lines, we indicated the real position of the tactile input in relation to the sound that is set to 0. Following a macro qualitative assessment, we can infer that all participants, regardless of age, exhibit a bias toward the hand following tactile sensory modality. This bias is also reflected in the bimodal conditions in which a ventriloquist-like effect is present because the bias is influenced by the actual location of the tactile stimulus, but it all seems to be shifted toward the hand. One might speculate that this attraction to the hand may be due to the hand being internalized as a prior, due to greater “usability”. Surely, this effect should be investigated in future studies.

Figure 2S. Bias for each bimodal condition, age-group, and group of participants (darker bars sighted, lighter bars blind). The lines represent the bias in the uni-sensory conditions for sighted (continuous lines) and blind (dashed lines). Error bars represent ± s.e.m.


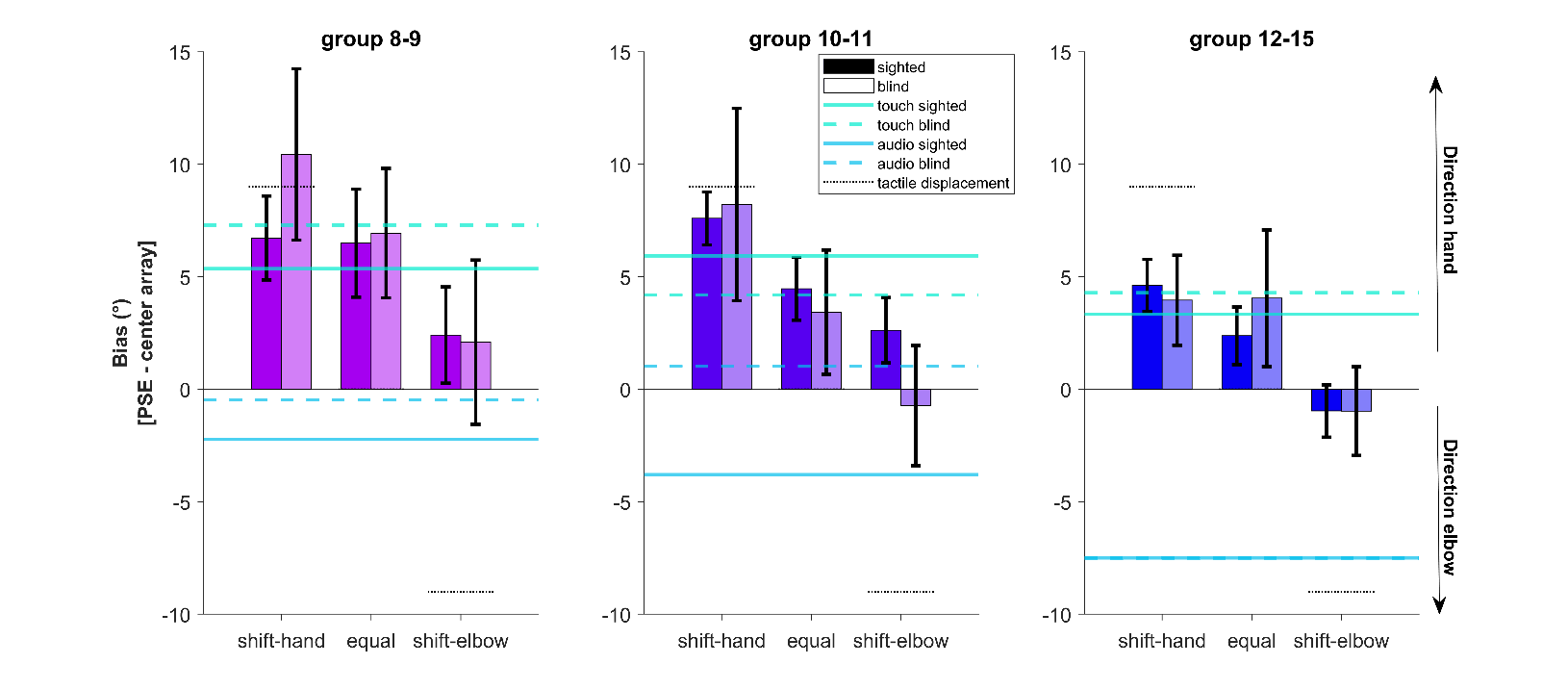


**Supplementary References**

1. Scheller, M. & Nardini, M. Correctly establishing evidence for cue combination via gains in sensory precision: Why the choice of comparator matters. *Behav Res Methods* (2023) doi:10.3758/s13428-023-02227-w.

2. Scarfe, P. Experimentally disambiguating models of sensory cue integration. *J Vis* **22**, 5 (2022).
